# Supplementary material for: A simple and effective F0 knockout method for rapid screening of behaviour and other complex phenotypes
Source: eLife. 2021 Jan 8;10:e59683. doi: 10.7554/eLife.59683 (PMC7793621; doi:10.7554/eLife.59683)
Supplement: Transparent reporting form [file elife-59683-transrepform.docx]

***eLife’s* transparent reporting form**

We encourage authors to provide detailed information *within their submission* to facilitate the interpretation and replication of experiments. Authors can upload supporting documentation to indicate the use of appropriate reporting guidelines for health-related research (see [EQUATOR Network](http://www.equator-network.org/%20)), life science research (see the [BioSharing Information Resource](https://biosharing.org/)), or the [ARRIVE guidelines](http://www.plosbiology.org/article/info:doi/10.1371/journal.pbio.1000412) for reporting work involving animal research. Where applicable, authors should refer to any relevant reporting standards documents in this form.

If you have any questions, please consult our Journal Policies and/or contact us: [editorial@elifesciences.org](mailto:editorial@elifesciences.org).

**Sample-size estimation**

- You should state whether an appropriate sample size was computed when the study was being designed
- You should state the statistical method of sample size computation and any required assumptions
- If no explicit power analysis was used, you should describe how you decided what sample (replicate) size (number) to use

Please outline where this information can be found within the submission (e.g., sections or figure legends), or explain why this information doesn’t apply to your submission:

Sample sizes were not computed by power analysis during experimental design, but are in line with typical sample sizes used for these experiments in larval zebrafish. Sample sizes (n, typically numbers of animals) are reported in text and/or in figures and/or in figure legends.

**Replicates**

- You should report how often each experiment was performed
- You should include a definition of biological versus technical replication
- The data obtained should be provided and sufficient information should be provided to indicate the number of independent biological and/or technical replicates
- If you encountered any outliers, you should describe how these were handled
- Criteria for exclusion/inclusion of data should be clearly stated
- High-throughput sequence data should be uploaded before submission, with a private link for reviewers provided (these are available from both GEO and ArrayExpress)

Please outline where this information can be found within the submission (e.g., sections or figure legends), or explain why this information doesn’t apply to your submission:

In phenotype-scoring experiments (Figure 1C,D,H,I; Figure 4A,C; Figure 1—figure supplements 1 and 2), progeny from different clutches (different parents) were scored and results pooled, thereby controlling partially for biological variability. For the Illumina MiSeq, 3–4 larvae (from the same clutch) were processed for each gene, providing biological replicates. Some results are replicates of each other: Figure 1C,D (number of loci = 3), Figure 1—figure supplement 1 (Cas9:gRNA ratio = 1:1), Figure 4A,B (*slc24a5*, *tyr*) are all technical and biological replicates of the same experiment; Figure 3B (uninjected, loci A, B, C) and Figure 3—figure supplement 1 (Phusion) are technical replicates of the same headloop PCRs (different reaction, gel). In Figure 5A,B and Video 3, animals from different clutches (different parents) were pooled, controlling partially for biological variability. The mustard oil assay (Video 3) was also replicated with different animals. Figure 6—figure supplement 1 (*scn1lab* F0 experiment 2) is a biological (different clutch from different parents) and technical (different ZebraBox) replicate of Figure 6 (*scn1lab* F0 experiment 1). Any animal excluded from analysis is reported and justified in Materials and Methods.

Illumina MiSeq data are available in the Zenodo repository: [10.5281/zenodo.3898915](https://doi.org/10.5281/zenodo.3898915)

**Statistical reporting**

- Statistical analysis methods should be described and justified
- Raw data should be presented in figures whenever informative to do so (typically when N per group is less than 10)
- For each experiment, you should identify the statistical tests used, exact values of N, definitions of center, methods of multiple test correction, and dispersion and precision measures (e.g., mean, median, SD, SEM, confidence intervals; and, for the major substantive results, a measure of effect size (e.g., Pearson's r, Cohen's d)
- Report exact p-values wherever possible alongside the summary statistics and 95% confidence intervals. These should be reported for all key questions and not only when the p-value is less than 0.05.

Please outline where this information can be found within the submission (e.g., sections or figure legends), or explain why this information doesn’t apply to your submission:

Data from individual animals were plotted wherever graphically possible to do so, with a black cross marking the population mean in scatter plots. If data from a population had to be summarised for plotting, data are plotted as mean ± standard deviation (SD) or standard error of the mean (SEM), which is reported in figure legends. Statistical tests and method of multiple test correction, sample sizes, dispersion and precision measures are reported in text or in figure legends. Statistical tests and method of multiple test correction are also summarised in Materials and Methods (section *Statistics)*. Exact p-values are reported in figure legends, or in text if the data were not plotted. Any p-value smaller than 0.001 is reported as p < 0.001.

(For large datasets, or papers with a very large number of statistical tests, you may upload a single table file with tests, Ns, etc., with reference to sections in the manuscript.)

**Group allocation**

- Indicate how samples were allocated into experimental groups (in the case of clinical studies, please specify allocation to treatment method); if randomization was used, please also state if restricted randomization was applied
- Indicate if masking was used during group allocation, data collection and/or data analysis

Please outline where this information can be found within the submission (e.g., sections or figure legends), or explain why this information doesn’t apply to your submission:

Allocations into groups (eg. F0 knockout vs scrambled-injected) occurred during injection of the eggs, and so was random. All scoring of phenotypes was performed blinded to the condition (eg. which Cas9:gRNA ratio): directly after injections, the Petri dish lids were covered with black tape to mask the annotations. The black tape was removed at the end of the experiment before euthanasia.

**Additional data files (“source data”)**

- We encourage you to upload relevant additional data files, such as numerical data that are represented as a graph in a figure, or as a summary table
- Where provided, these should be in the most useful format, and they can be uploaded as “Source data” files linked to a main figure or table
- Include model definition files including the full list of parameters used
- Include code used for data analysis (e.g., R, MatLab)
- Avoid stating that data files are “available upon request”

Please indicate the figures or tables for which source data files have been provided:

Source data and code are available at [github.com/francoiskroll/f0knockout](https://github.com/francoiskroll/f0knockout) and as a Zenodo repository: [10.5281/zenodo.3898915](https://doi.org/10.5281/zenodo.3898915)

Both repositories are the same, except for some heavy files which could not be uploaded directly to GitHub.
